# Supplementary material for: A Targeted and Tuneable DNA Damage Tool Using CRISPR/Cas9
Source: Biomolecules. 2021 Feb 15;11(2):288. doi: 10.3390/biom11020288 (PMC7919286; doi:10.3390/biom11020288)
Supplement: Supplementary file 1 [file biomolecules-11-00288-s001.pdf]

A targeted and tuneable DNA damage tool using CRISPR/Cas9.

Ioannis Emmanouilidis, Natalia Fili, Alexander W. Cook, Yukti Hari-Gupta, Ália dos Santos, Lin Wang, Marisa Martin-Fernandez, Peter J.I. Ellis and Christopher P. Toseland

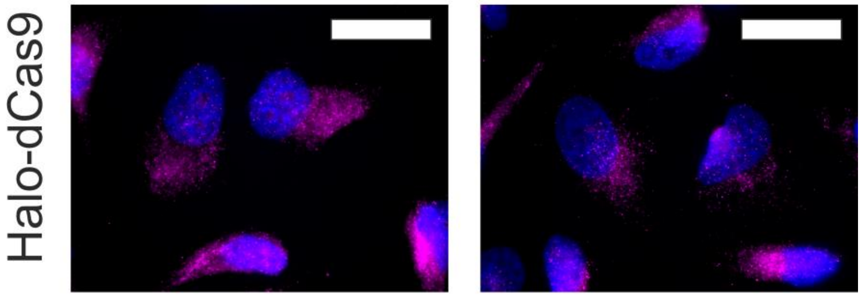

Supplementary Figure S1. Electroporation of HeLa cells. Example widefield image of Halo-dCas9 in HeLa cells stained with TMR ligand (magenta) and Hoechst for DNA (blue). Scale bar is 15 µm.

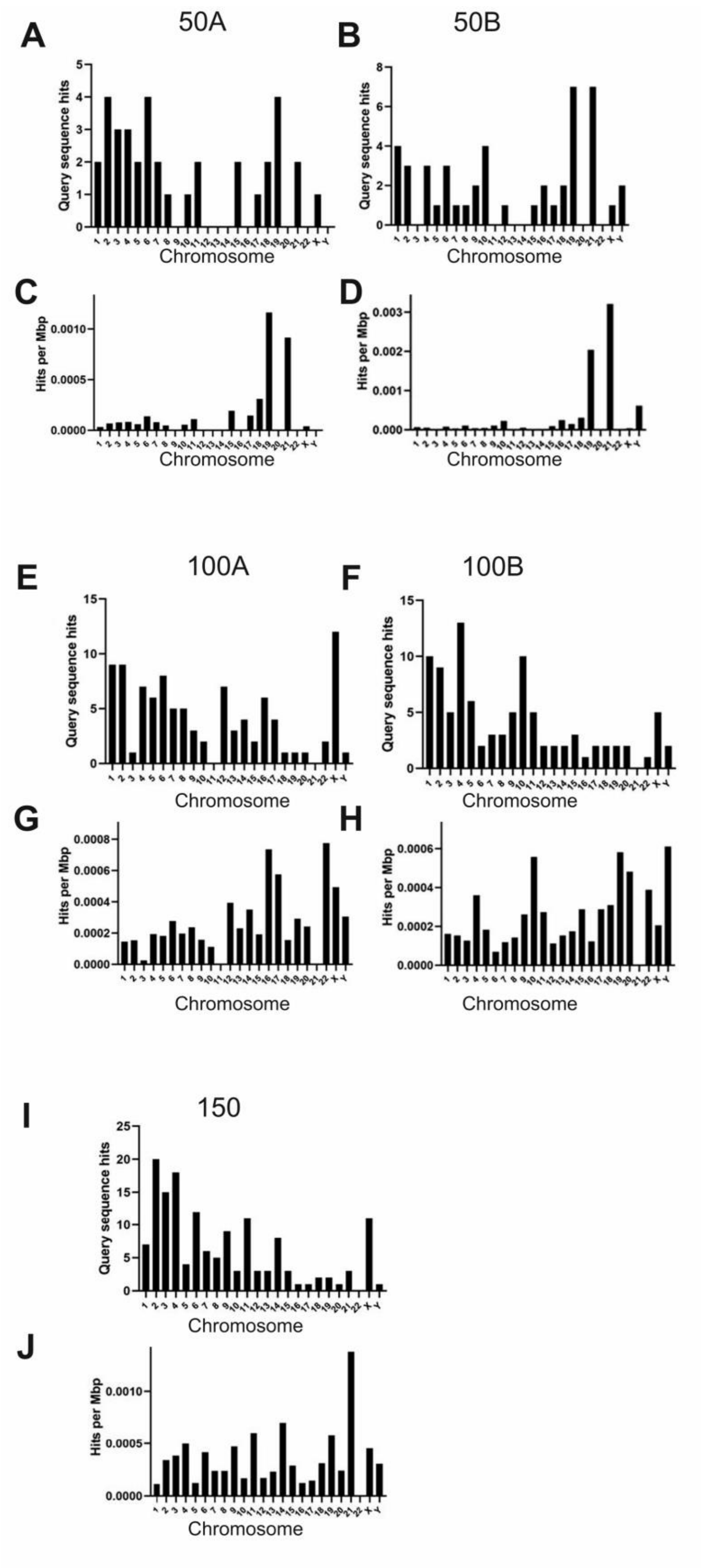

Supplementary Figure S2. Overview of genomic targeting for the crRNA sequences. Data are presented as Number of sequence hits (potential cuts) across the chromosomes and Hits per Mbp across the chromosomes for each of the crRNA guides designed in this study.

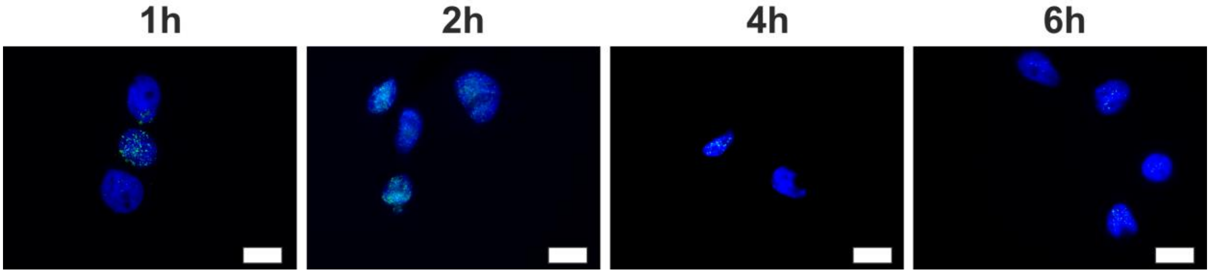

Supplementary Figure S3. Time course of Cas9 in MCF10a cells following electroporation. Immunofluorescence staining against Cas9 (green) in MCF10a cells. DNA was stained with Hoechst (blue). The time points refer to hours post-electroporation. Scale bar is 10 µm.
